# Spontaneous Cerebrospinal Fluid Rhinorrhea With Meningoencephalocele Recurrence After Placement of a Lumboperitoneal Shunt: A Case Report

**DOI:** 10.7759/cureus.58896

**Published:** 2024-04-24

**Authors:** Hiroki Natsuhara, Hideo Chihara, Isao Ono

**Affiliations:** 1 Neurosurgery, Hikone Municipal Hospital, Hikone, JPN

**Keywords:** lumbar drainage, lumboperitoneal shunt, cerebrospinal fluid diversion, cerebrospinal fluid rhinorrhea, meningoencephalocele

## Abstract

Cerebrospinal fluid rhinorrhea associated with meningoencephalocele is usually treated surgically. During the perioperative period, cerebrospinal fluid diversion may be employed to control intracranial pressure, but there are few indications for this method. A 51-year-old female presented with cerebrospinal fluid rhinorrhea associated with meningoencephalocele and underwent surgical repair followed by the placement of a lumboperitoneal shunt. However, cerebrospinal fluid leakage recurred, requiring a second surgery. Lumbar drainage effectively controls intracranial pressure, but it does not cure bone defects. The use of these devices should be carefully considered based on the patient's condition.

## Introduction

Cerebrospinal fluid (CSF) rhinorrhea can occur as a result of trauma, such as skull base fractures, iatrogenic conditions following neurosurgery, or nontraumatic conditions, such as infections or tumors [1]. Natural onset is rare, and some cases are attributed to meningoencephalocele [2]. Fundamentally, the recommended treatment for CSF leakage involves closing the leakage site to prevent complications such as meningitis. CSF diversion is often required during the postoperative period for augmentation of the surgical repair. However, there is insufficient evidence on the optimal timing for these procedures [3]. We present a case of spontaneous CSF leakage associated with meningoencephaloceles requiring reoperation after bone defect closure and placement of a lumboperitoneal shunt (LP shunt).

## Case presentation

**Figure 1:**
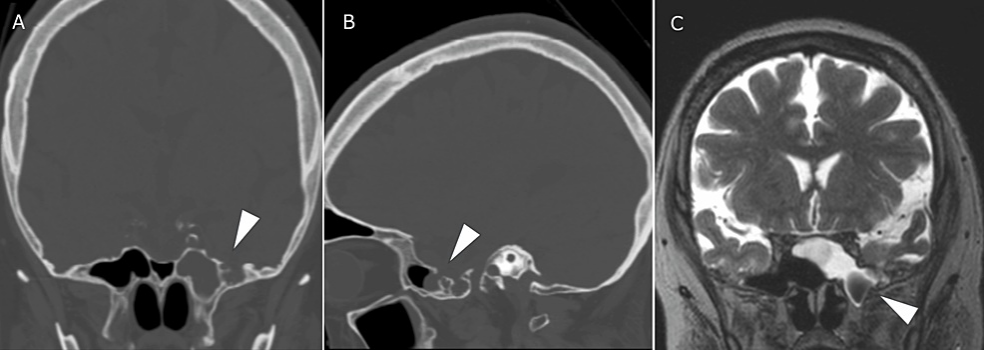
The CT and MRI scans show meningoencephalocele protruding into the left sphenoid sinus through the bone defect and CSF in the sphenoid sinus. Head CT showing a bone defect (arrowheads, A and B) in the left middle cranial fossa communicating with the sphenoid sinus. MRI showing meningoencephalocele in the sphenoid sinus (arrowhead, C) and accumulated cerebrospinal fluid within the sphenoid sinus. CT: computed tomography; MRI: magnetic resonance imaging; CSF: cerebrospinal fluid

A 51-year-old woman presented with intermittent watery rhinorrhea and headaches that persisted for more than a month. The patient was initially treated for chronic sinusitis, but further examination of nasal discharge components suggested CSF rhinorrhea. The patient had no history of head injuries or surgery. She was overweight, with a body mass index of 28.4 kg/m². Head computed tomography (CT) revealed a bone defect in the lateral wall of the left sphenoid sinus outside the foramen rotundum. Additionally, the findings included an empty sella and bilateral thinning of the middle cranial fossa. Magnetic resonance imaging (MRI) revealed a deviation of the brain parenchyma into the left sphenoid sinus (Figure 1A-C). A lumbar puncture revealed clear CSF, and the pressure was normal at 110 mmH_2_O.

**Figure 2:**
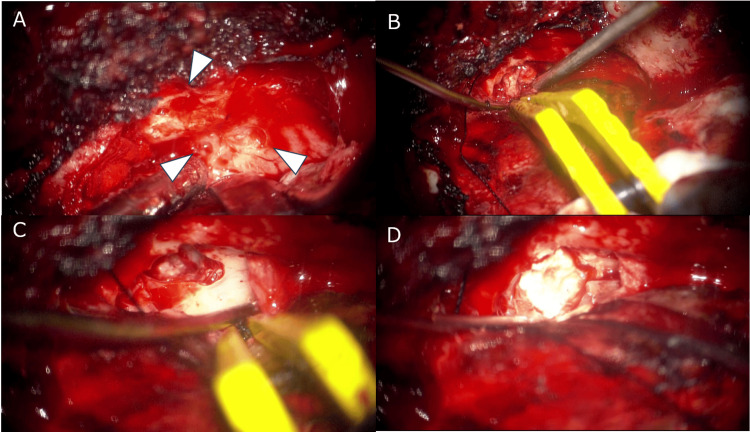
A left frontotemporal craniotomy was performed, and meningoencephalocele was excised approaching the extra dural side. Multiple bone pores (arrowheads, A) are observed, possibly related to thinning of the middle cranial fossa. Brain tissue is observed deviating from the bone defect and is cauterized (B and C). Repair of the bone defect using surgical bone cement (D).

Due to the suspicion that the meningoencephalocele was the underlying cause of the CSF leak, surgical repair was planned. A left frontotemporal craniotomy was performed. Approaching the middle cranial fossa on the extradural side, the brain parenchyma herniated from the dural defect was identified (Figure 2A-D). The fitted meningoencephalocele was excised, and the bone defect was closed with bone cement. The dura mater was closed using fibrin glue and biochemical reinforcement. CSF rhinorrhea initially stopped postoperatively but recurred 10 days later. Symptoms were temporarily relieved through bed rest and lumbar drainage. However, upon getting out of bed, CSF leakage recurred. Considering the potential for improvement through CSF management, an LP shunt was placed, and CSF rhinorrhea was ameliorated.

**Figure 3:**
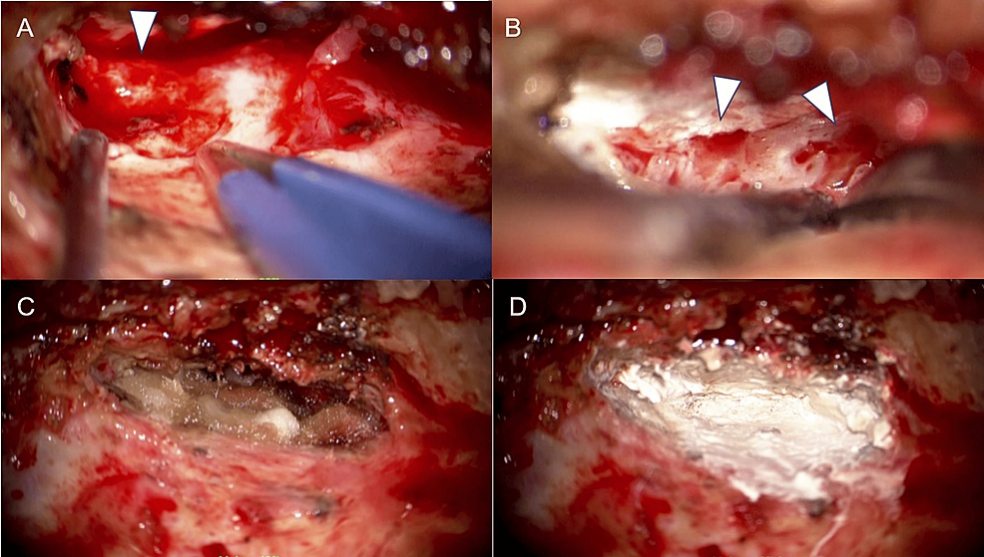
During the second surgery, residual bone defects and surrounding bone pores were observed and closed. The previously identified dural defect remained open, allowing the leakage of CSF (A). There were bony pores around the leakage point (arrowheads, B). The dural defect was closed using temporal fascia and fibrin glue (C). The middle cranial fossa was extensively repaired with bone cement while also covering the surrounding bone pores (D). CSF: cerebrospinal fluid.

However, three months later, she had a seizure, and her consciousness was suddenly impaired. A head CT scan revealed pneumocephalus. The symptom was considered to be caused by an incomplete closure of the bone and dural defect and the presence of an LP shunt. Therefore, a reoperation was performed. CSF leakage was identified at the site where the dural defect was sealed previously (Figure 3). The intraoperative findings suggested that the previous bone defect had been closed, but the bony pores may be involved in a current or future CSF leakage. The dura mater was closed using the same procedure as the initial surgery except for the use of temporal fascia. Bone cement was applied in sufficient quantity over the entire accessible left middle cranial fossa. After the second repair, CSF leakage stopped, and there was no recurrence for two years.

## Discussion

Pathogenesis

Patients with spontaneous CSF leakage are commonly linked to congenital defects of the dura mater and idiopathic intracranial hypertension [1]. Common radiographic findings in these cases include an empty sella, arachnoid pits, and skull base thinning [4]. With meningoencephalocele, the dura mater surrounding the bone defects from which the meningoencephalocele protrudes is likely under pressure. Intracranial hypertension may render patients more prone to dural damage, leading to degeneration of the meninges and resulting in dural defects. These factors are considered to be important for preventing future CSF leakage. In this case, although imaging findings were common, the patient’s intracranial pressure was normal. The possibility of intracranial hypertension in the past cannot be ruled out, as the CSF leakage resulted in a decrease in pressure.

Surgical approach

Surgical treatments include transcranial or endoscopic endonasal approaches, which are selected according to the location of the bone defects [3]. Bone and dural defects can be reconstructed in a multilayered fashion using materials such as free pericranial grafts, pedicled pericranial and temporal muscular flaps, and biological tissue reinforcement with fibrin glue [3,5]. In this case, multiple materials were used to create a multilayered restoration. Unfortunately, CSF rhinorrhea recurred after the initial surgery. As with bone defects in meningoencephalocele, small bony pits may provide mechanical stimulation to the dura mater and contribute to CSF leakage. Therefore, during reoperation, in addition to completely repairing the bone defects with bone cement, the surrounding small bony pits were also repaired. Bone cement was found to be useful for ensuring the quality and durability of the reconstructed middle cranial fossa.

Pros and cons of CSF drainage

Treatment for CSF rhinorrhea caused by meningoencephalocele involves surgical closure because the bone defects do not close spontaneously. Conservative treatments such as lumbar drainage and bed rest have been proposed for preventing intracranial hypertension during the perioperative period [3,5]. In this case, lumbar-peritoneal shunt surgery was performed, but the benefits did not outweigh the risks. Shunt surgery for cerebrospinal fluid should not be performed readily, as it poses risks of ventriculitis and meningitis. CSF drainage should be limited to a temporary measure during the perioperative period.

## Conclusions

In this case, complete closure could not be achieved initially. Closure of not only the bone defect where the meningoencephalocele was herniating but also the surrounding bony perforations using a sufficient amount of bone cement was deemed crucial. Using CSF diversions during the perioperative period should be carefully considered based on the patient's condition. It should be noted that once surgery has been performed, it is difficult to determine if complete closure has been achieved. Shunt surgery for CSF should not be chosen without careful consideration.
